# Evaluation of Medial Meniscal Extrusion Using Radiography

**DOI:** 10.3390/jcm12165268

**Published:** 2023-08-13

**Authors:** Shohei Murata, Hiroaki Kijima, Kimio Saito, Hidetomo Saito, Takanori Miura, Manabu Akagawa, Hiroaki Tsukamoto, Kana Sasaki, Toshihito Ebina, Koji Nozaka, Naohisa Miyakoshi

**Affiliations:** 1 Akita Sports Arthroscopy Knee Group (ASAKG), 1-1-1 Hondo, Akita 010-8543, Japan; 2 Department of Orthopedic Surgery, Kakunodate General Hospital, 3 Iwase, Senboku 014-0394, Japan; 3 Department of Orthopedic Surgery, Akita University Graduate School of Medicine, 1-1-1 Hondo, Akita 010-8543, Japan

**Keywords:** diagnostic imaging, knee, medial meniscal extrusion, radiography, magnetic resonance imaging

## Abstract

Recently, there has been increasing interest in medial meniscal extrusion (MME), but few reports have evaluated MME via X-ray. In this study, the amount of MME and meniscal height at the medial border of the tibia were measured via X-ray with gradation processing. The extrusion length divided by the meniscal height yields the meniscal extrusion ratio, which was used as an index. In addition, the medial meniscal length of the part protruding from the medial border of the tibia on MRI was measured as an absolute value. Then, the correlation between the meniscal extrusion ratio and the amount of MME on MRI was examined, and there was a strong correlation between the meniscal extrusion ratio via X-ray and the amount of MME on MRI (correlation coefficient 0.860, *p* < 0.0001). The cut-off value of the meniscal extrusion ratio via X-ray for positive meniscal extrusion on MRI was 0.50, with an AUC of 0.9825, sensitivity of 0.9063, and specificity of 0.8663. From the present study, it was possible to measure the extrusion length and meniscal height via gradation processing, with X-ray and without MRI, and to calculate the meniscal extrusion ratio, which strongly correlates with the amount of MME on MRI.

## 1. Introduction

Medial meniscal extrusion (MME) is associated with knee pain [1] and medial collateral ligament edema [2], and it is also reportedly associated with the severity and progression of osteoarthritis of the knee [3]. Identifying the presence and severity of MME on diagnostic imaging is therefore very important for evaluating the pathological condition of the knee joint and considering treatment strategy. The presence or absence of MME can be very helpful in determining the cause of knee pain, especially if there is no obvious evidence of knee osteoarthritis on plain radiographs.

The meniscus absorbs hoop stress and disperses pressure, but when a medial meniscal posterior root tear (MMPRT) occurs, the hoop breaks down, so that it can no longer fulfil this function, resulting in MME [4,5]. MMPRT thus causes rapid cartilage deformation and osteoarthritis [6] and is a risk factor for idiopathic osteonecrosis [7]. The identification of MME in patients presenting with knee pain at as early a stage as possible is thus very useful for treating many forms of knee pain. In other words, if the findings of MME can be recognized in a more convenient way and suspicion of MMPRT can be reached using the presence of typical clinical symptoms, MRI can be performed immediately, and surgery for MMPRT can be performed, since early surgery for MMPRT can prevent progression to osteoarthritis of the knee.

MRI is the diagnostic tool generally used for MME, and positive MME is most commonly defined as an extrusion of meniscal tissue by ≥3 mm from the medial margin of the tibia [8]. However, MRI has the disadvantage that the number of tests that can be performed in a day is lower than for other tests, due to the time required for imaging compared to X-ray or CT. In our region, an appointment for a knee MRI has to be booked about three weeks in advance. MRI also has a disadvantage in terms of cost. Therefore, it is not practical to perform MRIs every few weeks to assess whether the MME is going to progress in cases such as those with mild MME.

Although there have been some reports of the use of diagnostic ultrasound to evaluate MME [9], not all facilities are able to use ultrasound in everyday practice. Moreover, MME measurement by ultrasonography may be inconsistent among examiners, as absolute values vary depending on the position and method of applying the ultrasound probe.

On the other hand, plain radiography is the gold standard for the diagnosis of knee pain, and most orthopedic surgeons will conduct plain radiography for almost every patient who presents with knee pain as part of the initial consultation [10]. Accordingly, if plain radiography could be used to evaluate MME, in addition to osteophytes and cartilage wear, this would be very useful for assessing the prognosis of arthritic change and the cause of pain at an early stage.

Despite this, however, almost no studies have addressed the diagnosis of MME on plain radiography. This is because many orthopedic surgeons assume that plain radiographs are meant only to depict bone. However, recently, digital radiographs have come to be able to also allow limited visualization of soft tissues, owing to the ease of processing gradations in digital radiographs. We thus report a method that is extremely useful for the accurate diagnosis of MME using only plain radiography.

## 2. Materials and Methods

**Figure 1:**
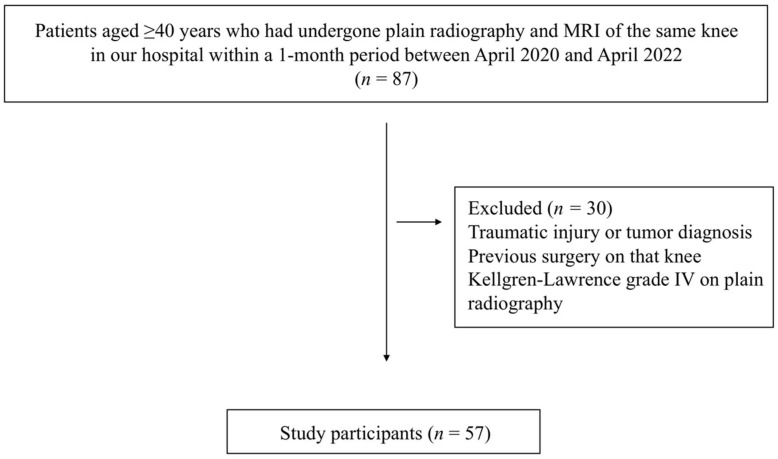
Study flow diagram.

The study subjects were patients aged ≥40 years who had presented to our hospital with a principal complaint of knee pain between April 2020 and April 2022 and who underwent both plain radiography and MRI within 1 month of presentation. Patients with knee pain due to traumatic injury, those diagnosed with a tumor, those who had previously undergone surgery on the knee under investigation, and those who were classified as Kellgren–Lawrence grade IV, with joint space narrowing that could render measurement difficult, were excluded (Figure 1). For the knees that met the inclusion criteria, patient age, sex, weight, and MME length measured on both MRI scans and gradation-processed radiographic images were investigated.

**Figure 2:**
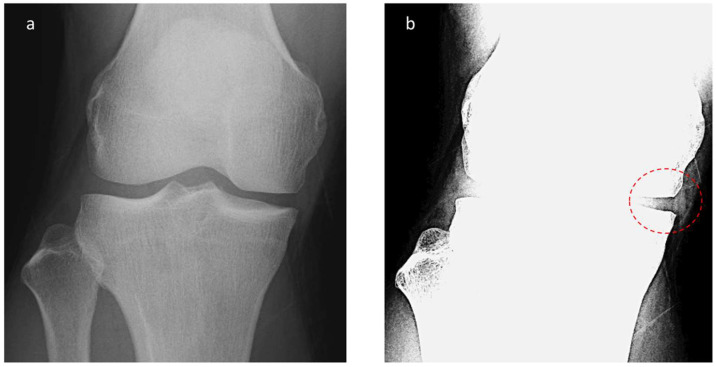
Radiographic appearance of meniscal extrusion. (**a**) Anteroposterior (AP) view knee radiograph with standard window settings. (**b**) AP knee radiograph with narrow window settings demonstrates visualized meniscal tissue.

**Figure 3:**
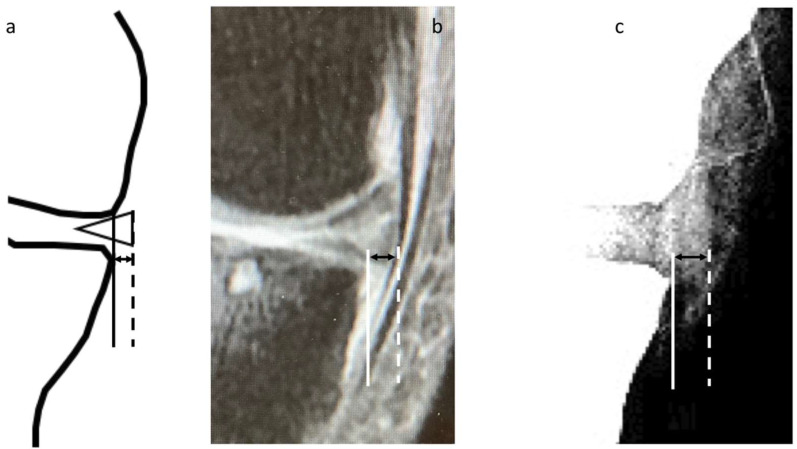
Measurement of meniscal extrusion. (**a**) A vertical line (solid line) is drawn intersecting the margin of the medial tibial plateau at the site of transition from horizontal to vertical. Extrusion is measured from this line to the outer edge of the meniscus (dotted line). (**b**) Measurement of the amount of medial meniscal extrusion on MRI (M-MME). (**c**) Measurement of the amount of medial meniscal extrusion on X-ray (X-MME).

**Figure 4:**
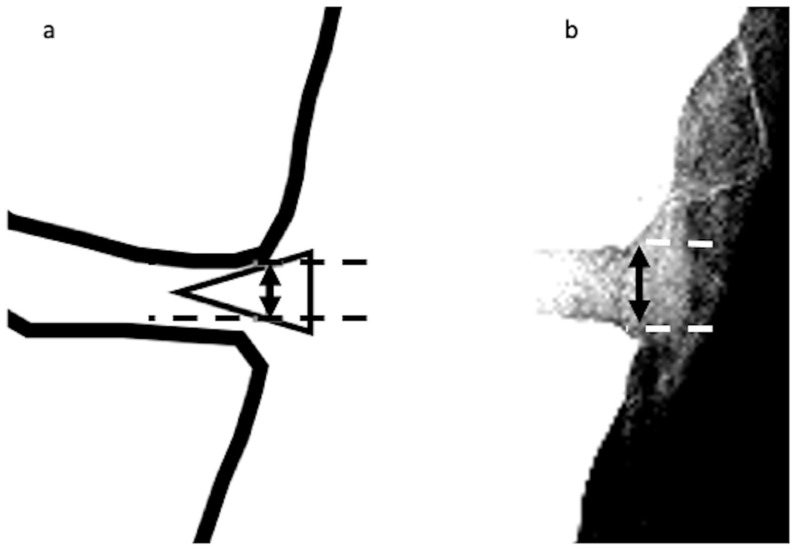
Height of the medial meniscus on X-ray (X-HMM). (**a**). A vertical line (solid line with arrowheads) is drawn intersecting the margin of the medial tibial plateau at the site of the transition from horizontal to vertical. The height is measured as the distance from the upper edge of the meniscus to the lower edge (dotted lines), as represented by the vertical line. (**b**) Measurement of X-HMM.

**Figure 5:**
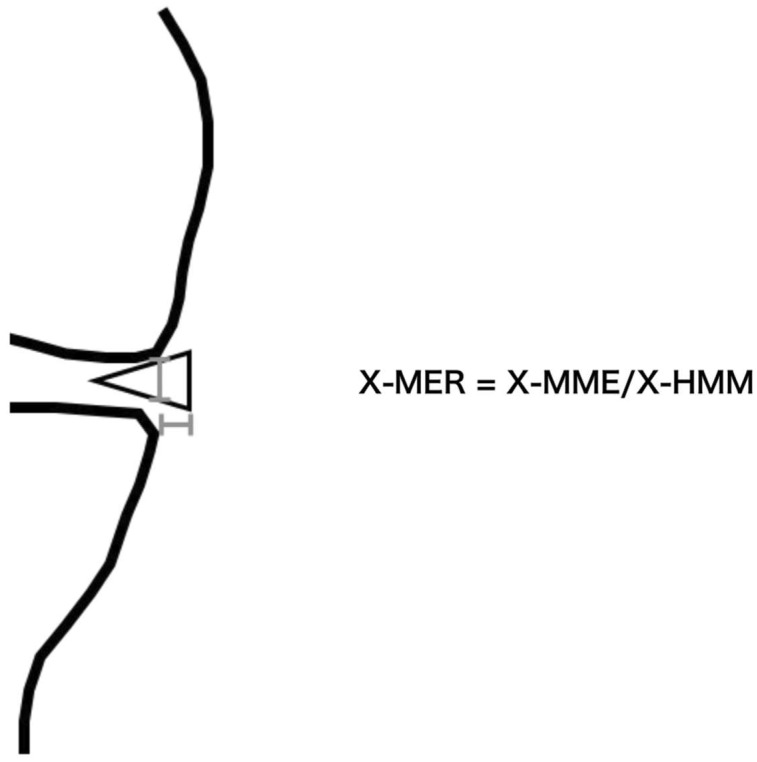
The meniscal extrusion ratio on X-ray (X-MER). The X-MER is defined as X-MME divided by X-HMM.

The MME length on radiographic images was investigated as follows. First, radiographs scanned in the knee anterior–posterior direction without weight bearing were subjected to appropriate window processing (specifically, narrowing the window width) to enable the medial meniscus to be more easily visualized (Figure 2). Next, the amount of MME (X-MME) from the medial margin of the tibia, excluding osteophytes (extrusion length) was measured (Figure 3), as was the height of the meniscus at the medial margin of the tibia, excluding osteophytes on X-ray (X-HMM) (Figure 4). Finally, the meniscal extrusion ratio was calculated by dividing the meniscal extrusion length by the meniscal height (X-MER) (Figure 5). Prior to these measurements, a small workshop to ascertain the appropriate gradation processing of radiographic images for use in meniscal measurements was conducted, using a radiology workstation (Synapse^®^ Cardiovascular Picture Archiving and Communication System (PACS) (Fujifilm, Tokyo, Japan)). After the appropriate gradation processing method for measurements of MME length and meniscal height had been confirmed, two orthopedic surgeons specializing in the knee each made two sets of measurements, and intra-investigator reliability and inter-investigator reliability were calculated.

Measurements of the amount of MME on MRI (M-MME) (Figure 2) were made via the method reported by Costa et al. [8]. MRI studies were performed using a 1.5-T machine (Signa Explorer 1.5T; GE Healthcare, Milwaukee, WI, USA). T2* (T2-star)-weighted images with a slice thickness of 3 mm taken at 15 degrees of knee flexion and no load were used for the measurements. The amount of MME was measured on a coronal image passing through the midpoint of the medial femoral condyle articular surface. Because that study reported that this method is highly reliable, with an intra-class correlation (ICC) of 0.970 [8], it was not necessary to confirm the ICC of the M-MME in the present study.

The sample size was set to provide statistical power of at least 80% (α = 0.05), and after linear regression was conducted, Pearson’s correlation coefficient was calculated. With positive meniscal extrusion defined as a meniscal extrusion length ≥ 3 mm on MRI images, a receiver-operating characteristic (ROC) curve for the association between this and X-MER was created, and the cut-off value of X-MER for positive meniscal extrusion that maximized sensitivity and specificity was calculated. In all statistical tests, *p* < 0.05 was considered significant. EZR (Saitama Medical Center, Saitama, Japan), a graphical user interface for R (R Foundation for Statistical Computing, Vienna, Austria), and JMP (JMP ver.14.2, SAS Institute, Cary, NC, USA) were used as statistical software.

This research was conducted in accordance with the guidelines of the Declaration of Helsinki and was approved by the authors’ institutional review board (approval no. 420). Since it involved the retrospective use of anonymized data obtained for clinically necessary purposes, written, informed consent from individual patients was not required.

## 3. Results

A total of 57 knees of 57 patients (15 men and 42 women; mean age 72 years; mean weight 68.1 kg) were evaluated. Of these, 30 knees were Kellgren–Lawrence grade I, 18 were grade II, and 9 were grade III (Figure 1, Table 1). The mean M-MME was 3.45 ± 1.80 mm, and meniscal extrusion ≥ 3 mm on MRI was observed in 32 knees.

**Figure 6:**
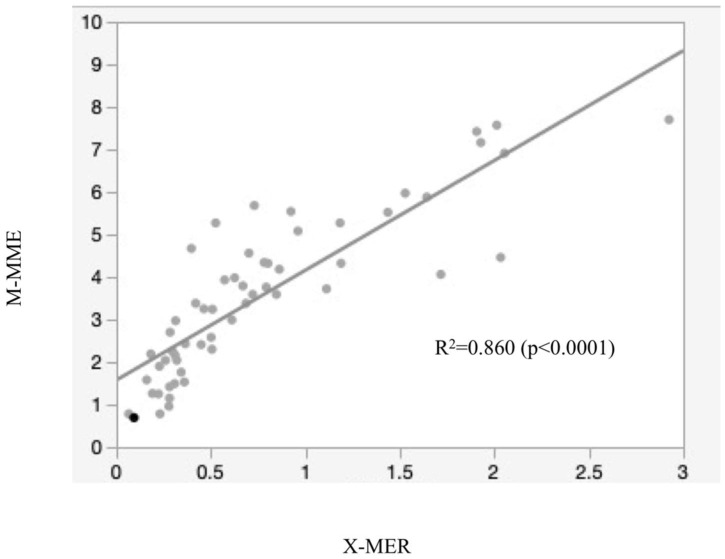
Correlation between X-MER and M-MME (Pearson’s correlation coefficient R^2^ = 0.860).

In terms of the reliability of X-MER, the intra-class correlation coefficient was 0.983 for Rater 1 and 0.946 for Rater 2 (Table 2), the inter-class correlation coefficient was 0.854 (95% confidence interval 0.766–0.911), and the mean value of X-MER was 0.755 ± 0.624 (Table 1). X-MER was strongly correlated with M-MME (correlation coefficient 0.860, *p* < 0.0001) (Figure 6).

**Figure 7:**
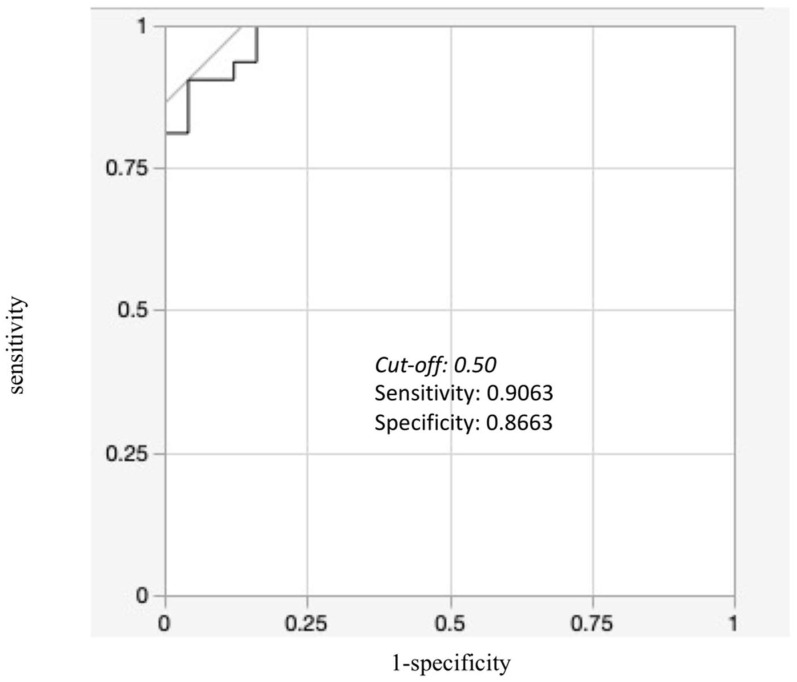
Receiver-operating characteristic (ROC) curve of X-MER for M-MME greater than 3 mm. The cut-off value of X-MER for MME greater than 3 mm is 0.50, with sensitivity of 0.9063 and specificity of 0.8663.

With positive meniscal extrusion defined as an M-MME ≥ 3 mm on MRI, an ROC curve was created with X-MER as the predictor variable, and with a cut-off value of X-MER of 0.50 for positive meniscal extrusion on MRI, the area under the curve was 0.9825, sensitivity was 0.9063, and specificity was 0.8663 (Figure 7).

## 4. Discussion

Very few studies have previously reported the feasibility of diagnosing MME on plain radiography, but the present results suggest that performing gradation processing of plain radiographs enables meniscal extrusion to be detected at an equivalent level to M-MME.

The Appropriateness Criteria of the American College of Radiology (ACR) [10] recommend radiography as the most appropriate initial investigation for non-traumatic knee pain, and most orthopedic surgeons conduct plain radiography as part of the initial consultation for almost all patients with knee pain. Radiographic images can show not only osteophytes and the severity of cartilage wear, but also other factors, such as osteonecrosis in the femoral condylar area, tumorous lesions, overall knee alignment, and the condition of the patello-femoral joint, and they are also a suitable aid for explaining the disease to the patient [11,12,13]. In most cases, however, in the absence of obvious abnormalities, knee pain is normally due to early-stage osteoarthritis, and MME is one finding that has been found to be associated with such pain [14]. This means that the ability to determine whether MME is present on plain radiography, the first investigation carried out during initial consultations, would enable the correct treatment to be started at a very early stage.

The only previous study of the detection of MME on plain radiographs simply compared the association between the qualitative assessment of its presence on radiographic and MRI images [15]. In that study, after MME had been made more visible on radiographs via gradation processing, two orthopedic surgeons measured the amount of MME, defined MME greater than 3 mm as positive, and compared the agreement with MRI. In that report, there was no mention of X-ray methods or methods of correcting for scale. On radiographs, however, the reduction scale varies depending on the distance between the radiography unit and the scanning plane when the scan is taken, and it is impossible to assess meniscal extrusion on general radiographs by measuring the absolute amount of extrusion in the same way as on MRI scans. It is thus impossible to use this method to measure MME in routine practice or for determining MME cut-off values.

In the present study, whether it was possible to detect meniscal extrusion on radiographs by measuring the relative amount of meniscal extrusion and the relative meniscal height and calculating the meniscal extrusion ratio was investigated. The association between the meniscal extrusion ratio on radiographs and the amount of meniscal extrusion on MRI was then evaluated. This is the first study to calculate a cut-off value for the meniscal extrusion ratio, a value that can be quantitatively measured, on plain radiographs.

The use of this method could enable the detection of meniscal extrusion on routine knee radiographic images, with far higher accuracy than that reported in the above-mentioned qualitative study, by using the appropriate window settings and measuring the amount of meniscal extrusion and meniscal height. Radiography offers the most appropriate starting point for the imaging assessment of knee pain, and if the presence of meniscal extrusion could be detected radiographically during the initial consultation, this would enable the cause of the knee pain to be inferred, as well as facilitate consideration of the necessity of MRI or other additional imaging investigations and careful follow-up. Specifically, based on the results of the present study, in cases where the meniscal extrusion ratio exceeds 0.50 on radiography, and the patient complains of severe posterior medial knee pain, the presence of an MMPRT lesion can be suspected, and MRI can be scheduled as a priority. In addition, the meniscal extrusion ratio correlates well with the amount of meniscal extrusion on MRI, making it possible to assess the presence and progression of MME using knee radiographs taken for follow-up before and after knee surgery. In addition, since this method can be used for previous radiographs not taken under special conditions, in patients currently presenting with knee osteoarthritis, previous images, if available, can be used to investigate whether and to what extent MME was present before the onset of osteoarthritis. The present results showed high intra-rater and inter-rater reliability, with the ICC exceeding 0.7 in all cases, a level of reliability that makes this method usable in everyday clinical practice.

Ultrasonography can also facilitate the immediate measurement of the amount of medial meniscal extrusion as the absolute value, as in MRI. However, unlike in MRI or plain radiographs, there may be a considerable measurement error in the amount of MME depending on the position and method of application of the ultrasound probe.

On the other hand, since the plain radiographs themselves are highly reproducible, if the knee position at the time of imaging is standardized using a brace, etc., and if the gradation processing, measurement, and calculation of the meniscal extrusion ratio are automated using artificial intelligence, a system can be created in which the presence and degree of MME can be communicated to the attending physician as numerical values simply by taking X-rays, thus making plain radiographs a marker for quantitative osteoarthritis of the knee. This system would be more commonly used as a quantitative marker for osteoarthritis of the knee.

One limitation of the present study is that it was not possible to investigate the association with actual meniscal lesions. The vast majority of study subjects did not undergo subsequent arthroscopy. Further studies including more patients using this method together with arthroscopy might enable the calculation of a cut-off value for the detection of meniscal lesions.

In addition, the present study did not examine the amount of MME, the extrusion ratio, and the cut-off value for each osteoarthritis stage. By collecting and examining cases in the early stage of knee osteoarthritis, Kellgren–Lawrence I, it may be possible to calculate more clinically meaningful cut-off values of MME with respect to prevention of the onset and progression of knee osteoarthritis.

In addition, this study was conducted at a single facility, using a single X-ray machine, a single MRI machine, and a single radiology workstation. Further studies at more facilities and with more types of equipment will validate the accuracy of this method more universally.

The present study also used non-weight-bearing plain radiographs to enable comparisons with the results of non-weight-bearing MRI scans. However, meniscal extrusion is known to be more obvious on weight-bearing radiographs, and it may therefore be possible to adapt this method for use with weight-bearing plain radiographs and conduct further studies of more patients to calculate a cut-off value with a direct clinical linkage equal to or greater than that of M-MME. Thus, the present study provides important evidence that constitutes the first step toward the development of more useful ways of making use of plain radiography, as described above.

## 5. Conclusions

Radiography is the first-choice diagnostic imaging modality for patients complaining of knee pain, but few studies have addressed the use of radiographs for the evaluation of MME. The present results showed that with a cut-off value for X-MER of 0.50, meniscal extrusion was detectable with high sensitivity and specificity without the need for MRI scanning.

## Figures and Tables

**Table 1:** Patients’ characteristics.

No. of patients/knees	57 (57 knees)
Sex, male/female	15/32
Age (y)	72 ± 13 (45–82) *
Weight (kg)	68.1 ± 14.7 **
K-L grade	I: 30, II: 18, III: 9
M-MME (mm)	3.45 ± 1.80 **
M-MME > 3 mm	32 knees
X-MER	0.755 ± 0.624 **

* mean (range). ** mean ± standard deviation. K-L grade: Kellgren–Lawrence grade.

**Table 2:** Intra-rater correlations and 95% confidence intervals.

Rater	Intra-Class Correlation Coefficient	95% Confidence Interval
1	0.983	0.971–0.989
2	0.946	0.911–0.968

## Data Availability

The majority of data analyzed in this study are presented in the Section 3. Further data are available from the corresponding author upon reasonable request.
